# African ancestry is associated with facial melasma in women: a cross-sectional study

**DOI:** 10.1186/s12881-017-0378-7

**Published:** 2017-02-17

**Authors:** Maria Paula Barbieri D’Elia, Marcela Calixto Brandão, Bruna Ribeiro de Andrade Ramos, Márcia Guimarães da Silva, Luciane Donida Bartoli Miot, Sidney Emanuel Batista dos Santos, Hélio Amante Miot

**Affiliations:** 1 Department of Dermatology, FMB-Unesp, Botucatu, SP Brazil; 2 Department of Pathology, FMB-Unesp, Botucatu, SP Brazil; 3 Institute of Biological Sciences, UFPA, Belém, PA Brazil

**Keywords:** Melasma, Melanosis, Contraceptives, Oral contraceptives, Pregnancy, Hormones, Gonadal steroid hormones, Melanosis, Pigmentation, Skin pigmentation, Ultraviolet rays, Pigmentation disorders, Ancestry, INDEL

## Abstract

**Background:**

Melasma is a chronic acquired focal hypermelanosis affecting photoexposed areas, especially for women during fertile age. Several factors contribute to its development: sun exposure, sex steroids, medicines, and family history. Melanic pigmentation pathway discloses several SNPs in different populations. Here, we evaluated the association between genetic ancestry and facial melasma.

**Methods:**

A cross-sectional study involving women with melasma and an age-matched control group from outpatients at FMB-Unesp, Botucatu-SP, Brazil was performed. DNA was extracted from oral mucosa swabs and ancestry determined by studying 61 INDELs. The genetic ancestry components were adjusted by other known risk factors by multiple logistic regression.

**Results:**

We evaluated 119 women with facial melasma and 119 controls. Mean age was 39 ± 9 years. Mean age at beginning of disease was 27 ± 8 years. Pregnancy (40%), sun exposure (37%), and hormonal oral contraception (22%) were the most frequently reported melasma triggers. All subjects presented admixed ancestry, African and European genetic contributions were significantly different between cases and controls (respectively 10% vs 6%; 77% vs 82%; *p* < 0.05). African ancestry (OR = 1.04; 95% CI 1.01 to 1.07), first generation family history (OR = 3.04; 95% CI 1.56 to 5.94), low education level (OR = 4.04; 95% CI 1.56 to 5.94), and use of antidepressants by individuals with affected family members (OR = 6.15; 95% CI 1.13 to 33.37) were associated with melasma, independently of other known risk factors.

**Conclusions:**

Facial melasma was independently associated with African ancestry in a highly admixed population.

**Electronic supplementary material:**

The online version of this article (doi:10.1186/s12881-017-0378-7) contains supplementary material, which is available to authorized users.

## Background

Melasma is an acquired chronic hypermelanosis which mainly affects women during fertile age. Its aetiology is not fully understood, however, many factors can trigger or aggravate it: sun exposure, hormonal contraception (HC), hormone replacement therapy, cosmetics, photosensitising medication, pregnancy, and psychological stress. High levels of occurrence in families (40–60%) suggest a genetic component [1–3].

Ancestry-informative markers (AIMs) are used to estimate ancestral contributions to individuals due to their large allele frequency differences between geographically or ethnically defined populations [4]. AIMs have not been used to estimate the genetic contribution of population ancestry in melasma. However, self-reported Amerindian ancestry was linked to melasma in Brazil [5]. Some authors have also identified polymorphisms and different gene expression patterns related to skin pigmentation which are associated with the population origin [6–8].

The Brazilian population originated as a result of the admixture process that took place mostly between three ancestral groups, namely European colonisers, enslaved Africans and native Amerindians, which started more than 500 years ago [9–11]. This study aimed to evaluate genetic ancestry in Brazilian patients with facial melasma.

## Methods

A cross-sectional study was performed with women over 18 years-old under treatment at Botucatu University Hospital – UNESP between November/2013 and May/2014. The project was approved by the institutional research ethics committee.

Cases were determined by the presence of facial melasma, clinically confirmed by a dermatologist. Controls were paired with cases according to age group (±5 years).

Subjects were not included if they had other facial dermatoses, reported Asian descent, or extreme Fitzpatrick’s phototypes I and VI (due to the absence of these extreme phototypes in this sample of melasma patients).

Sampling was performed by convenience on consecutive patients seen at the clinic. Subjects were interviewed by the researcher using a semi-structured form, they then rinsed their mouths with clean bottled mineral water and a sample of oral mucosa was taken using a swab.

DNA was extracted using a QIAmp DNA micro kit in a QIAcube and the QIAmp® DNA Investigator kit protocol provided by QIAGEN (USA) [12], in the Maternal Fetal Laboratory – UNESP. The samples were amplified and genotyped for 61 insertion-deletion (INDEL) AIMs (Additional file 1: Table S1) valid for the Brazilian population in the Human Genetics and Medicine Laboratory (LGHM-UFPA) [13, 14].

Amplification was performed using a multiplex PCR technique in a final volume of 10 μL (1 μL DNA + 1 μL Primer mix + 5 μL Taq PCR Master Mix Qiagen + 1 μL Qsolution + 2 μL water) [14]. The PCR product (1 μL) was added to a mix containing 8.7 μL formamide and 0.3 μL Gene Scan 500 LIZ standard size ladder (Applied Biosystems). The DNA fragments were genotyped by capillary electrophoresis using an automatic ABI PRISM® 3130 Genetic Sequencer Analyser (Applied Biosystems, USA), and analysed with Gene Mapper® IDv3.2 (Applied Biosystems). Allele identification was performed with reference to the 500 LIZ standard size ladder (Applied Biosystems) [14].

The ancestry of the Brazilian samples was estimated using STRUCTURE v2.3.4, using three parental populations (Amazonian Amerind, Sub-Saharan African and Western European). This assessment was based on a validated database with 593 individuals of known ancestry that resulted in 98% correct classification: Sub-Saharan Africans - 189 individuals fromAngola, Mozambique, Zaire, Cameroon, and the Ivory Coast; Europeans −161 individuals, mainly Portuguese; and Amerindians - 243 individuals from indigenous tribes of the Brazilian Amazon region [14–17].

Categorical and ordinal data were shown as percentages and compared between groups by the chi-squared or chi squared for trend test. Continuous data were represented by means and standard deviations or medians and quartiles (p25–p75) according to the Shapiro-Wilk test (normality), and compared by the Student *t*, Mann-Whitney or Jonckheere-Terpstra tests [18, 19].

Correlations between ancestral components and other continuous variables were estimated by Spearman’s coefficient of correlation (rho) [18, 20].

Ancestry components were tabulated and percentages from each group compared between cases and controls or according to phototype or schooling by multivariate analysis of variance (*MANOVA-Pillai’s trace*) with *post-hoc* Bonferroni test. Ancestral components were normalised with a log_10_ transformation (for the MANOVA test). Homoscedasticity was evaluated with the Levene test [18].

In order to adjust the effect of ancestry for clinical and other epidemiologic factors, a multivariate model (conditional multiple logistic regression) was built. The covariates were included according to their significance (*p* < 0.2) at the bivariate analysis, but collinearities were supressed. Interactions between final terms of the model were tested for their additive effect. Effect size was estimated by odds ratio (OR) and its 95% confidence interval [21, 22].

Missing data (<10%), for multivariate analysis, were estimated by multiple imputation using ten iterations [23].

Sensitivity analysis was performed by: evaluating the final multivariate logistic regression model without imputed data.

The sample size calculation was based on a pre-test with 100 cases and 100 controls calculated for a final multiple logistic regression model with 80% power and a two-sided alpha level of 0.05. It resulted in 110 subjects in each group, leading to an effect size (R^2^) of more than 0.3 [21, 22, 24].

Data were analysed with IBM-SPSS 20.0 [25]. Significance was set at *p* < 0.05.

## Results

We evaluated 119 women with facial melasma and a similar number of controls (*n* = 238). Clinical characteristics of patients with facial melasma are shown in Table 1. The onset of melasma was during fertile age; pregnancy and sun exposure were the most prevalent triggering factors; and centrofacial topography was the most affected area. No melasma patient had skin phototype I or VI. Pregnancy occurred in 92 (77%) cases, and pregnancy-induced melasma was reported by 48 (52%) patients who had become pregnant.

**Table 1:** Clinical and demographic data for patients with melasma

Age of melasma onset (years)^a^	27.2 (8.3)
Reported trigger factor − *N* (%)	
Pregnancy	48 (40)
Sun exposure	44 (37)
HC	26 (22)
HRT	2 (2)
Cosmetics/Treatments	7 (6)
Stressful events	3 (3)
Others	6 (5)
Affected facial site − *N* (%)	
Zygomatic	60 (50)
Frontal	48 (40)
Upper lip	44 (37)
Temporal	39 (33)
Mentonian	25 (21)
Mandibular	22 (18)
Parotid	20 (17)
Nasal	19 (16)
Glabelar	15 (13)
Affected facial sites^b^	3 (2–4)

*Abbreviations*: *HC* hormonal oral contraception, *HRT* hormonal replacement therapy

^a^mean (st deviation)

^b^median (p25–p75)

Main demographic and genetic ancestry data for the groups are shown in Table 2. There is a higher frequency of first generation family report of melasma, history of pregnancy, daily sun exposure, low schooling level and African ancestry among cases. All subjects presented mixed genetic ancestry. European ancestry was predominant in both groups followed by African and Amerindian components.

**Table 2:** Demographic and genetic ancestry data from groups

Variables	Melasma (*n* = 119)	Controls (*n* = 119)	*Bivariate analysis*		*Multivariate analysis*	
			Odds Ratio (CI 95%)	*p*	Odds Ratio (CI 95%)	*p*
Age (years)^a^	39.0 (8.2)	39.0 (9.7)	–	–	0.99 (0.95–1.02)	0.44
Skin phototype-*N* (%)				0.09		0.13
II	18 (15)	30 (25)	1.00 (–)		1.00 (–)	
III	42 (35)	45 (38)	1.56 (0.76–3.20)		1.32 (0.57–3.02)	
IV	46 (39)	30 (25)	2.56 (1.22–5.38)		1.40 (0.58–3.39)	
V	13 (11)	14 (12)	1.55 (0.60–4.02)		0.39 (0.11–1.40)	
Education level-*N* (%)				<0.01		0.01
Elementary-Middle school	39 (33)	14 (12)	4.67 (2.24–9.73)		4.04 (1.62–10.11)	
High school	43 (36)	43 (36)	1.68 (0.93–3.01)		1.71 (0.85–3.44)	
College	37 (31)	62 (52)	1.00 (–)		1.00 (–)	
Family with melasma (first degree)-N (%)	67 (56)	27 (24)	3.10 (1.11–8.70)	0.03	3.04 (1.56–5.94)	<0.01
Age of menarche (years)^a^	12.8 (1.8)	12.7 (1.8)	1.01 (0.88–1.16)	0.92	–	–
Time using hormonal contraception (years)^b^	10 (3–15)	5 (1–12)	1.03 (0.98–1.07)	0.13	1.02 (0.98–1.07)	0.33
Daily regular sun exposition-*N* (%)	42 (44)	24 (27)	2.15 (1.16–3.99)	0.02	1.09 (0.57–2.10)	0.80
Pregnancy history-*N* (%)	92 (77)	77 (65)	1.86 (1.05–3.29)	0.03	1.50 (0.69–3.29)	0.31
Psycotropic drugs (regular use)-*N* (%)						
Antidepressant	28 (24)	18 (15)	1.73 (0.90–3.33)	0.10	2.96 (0.37–24.03)	0.31
Anxiolytic	11 (9)	5 (4)	2.32 (0.78–6.90)	0.12	–	–
Genetic ancestry (%)^b^						
European component	77 (64–89)	82 (69–90)	0.98 (0.97–0.99)	0.04	–	–
Amerindian component	7 (4–14)	7 (4–14)	1.00 (0.97–1.02)	0.72	–	–
African component	10 (4–21)	6 (4–16)	1.03 (1.01–1.05)	<0.01	1.04 (1.01–1.07)	<0.01
Interactions						
Antidepressant^a^ Family with melasma					6.15 (1.13–33.37)	<0.01
Antidepressant^a^ Pregnancy history					0.14 (0.01–1.35)	0.09

*p* (overall model) < 0.01; Hosmer-Lemeshow test: *p* = 0.98; Correct classification: 72%; R^2^ (Nagelkerke): 0.31

^a^mean (st deviation)

^b^median (p25–p75)

**Fig. 1:**
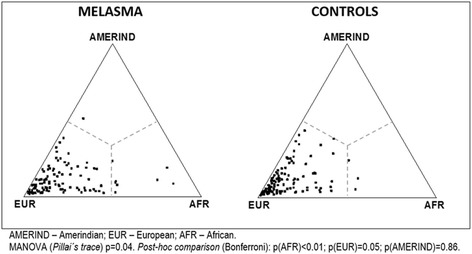
Ternary plot of group genetic ancestral composition

Figure 1 shows case and control distribution related to genetic ancestry. There was a significant difference between groups for ancestral components with significant variation for African and European ancestry.

Multivariate analysis (Table 2) showed that the following were independently significant for melasma development: low education level, family history of melasma and African ancestry. Significant interaction was seen between use of antidepressants and family history of melasma (OR = 6.15; 95% CI 1.13 to 33.37; *p* < 0.01).

Sensitivity analysis using logistic regression without imputed data (*n* = 180) provided a similar result to the complete model (Additional file 2: Table S2).

Subjects with higher education levels exhibited higher median European ancestry and lower Amerindian ancestry than those with lower education levels, without difference between cases and controls (*p* > 0.1). Estimated ancestral contributions according to college, high school and elementary education were for European: 84, 79 and 76% (*p* < 0.01); for African: 5, 9 and 11% (*p* < 0.01); and for Amerindian: 6, 7, 9% (*p* = 0.01).

There was moderate correlation between European (rho = −0.43; *p* < 0.01) and African (rho = 0.41; *p* < 0.01) ancestry component and phototypes, but poor correlation with phototype for the Amerindian ancestral component (rho = 0.18; *p* < 0.01) (Additional file 3: Table S3).

Among melasma patients, genetic ancestry did not show an association with reported triggering factors, family history, or age at disease onset. The number of affected facial topographies decreased in proportion to education level: mean 4.0, 3.4 and 2.4 lesions (Additional file 4: Table S4) among participants with elementary, high school and college education (*p* < 0.01). Similarly, it was weakly correlated with the European ancestral component (rho = −0.23; *p* = 0.01), but not with the African and Amerindian components (rho = 0.18 and 0.15; *p* > 0.05).

## Discussion

African genetic ancestry, as well as education and family history are associated with the development of female facial melasma in a highly genetically admixed population, independently of skin phototype and other risk factors.

Melasma patients in this study presented phenotype, clinical, and demographic characteristics similar to other Brazilian studies: disease onset during fertile age (16 to 35 years); the main triggers being sun exposure and hormones (pregnancy and HC), preferentially affecting the centrofacial region, high occurrence in family members and greater prevalence among dark phototypes (III to V) [3, 26].

Ancestral origin can be associated with diverse immunological responses patterns, metabolic processes, host response to infections, and drug treatment outcomes which justify clinical studies investigating ancestral components in admixed populations [27–31]. Amerindian ancestry is associated with a lower risk of leprosy [32] as well as for Alzheimer’s disease [33], but a higher risk of systemic lupus erythematous [34, 35]. European ancestry is associated with higher risk of neuromyelitis optica and multiple sclerosis, [36] sleep apnoea, [37] and death from heart failure [38]. Obesity shares African origin genes, [39] as do asthma and IgE level [40].

Brazil, especially the studied region, has experienced considerable admixture between Amerindian, European and African populations [41]. Skin pigmentation exhibits polygenic inheritance and several single nucleotide polymorphisms were identified in pigmentation genes in different ancestral groups [6–8, 42].

Despite the high admixture rate, European ancestry is the most prominent among the Brazilian population, as shown in our sample [11]. Physical traits and parental reports can lead to a mismatch between self-reported and genetic ancestry, which may explain the lack of association observed in our study between Amerindian ancestry and melasma, even though a positive association was described earlier in the same population [5, 43].

Melasma is less prevalent in Europeans, and more frequent in Asians and Latin Americans, who have a common phylogenetic origin in human migrations from Africa [44, 45]. In a study of genetic ancestry of the skin pigment system, similarities were seen between European and Asian expression patterns which differed from the African pattern. The authors also showed that pigmentation is the result of complex cellular interactions in which a large contingent of genes and regulatory factors are not completely understood, such as NINL, S100A4, H19, WIF-1, PDZK1, sex steroids, and miRNA-675 [46–52].

Furthermore, melanogenesis in melasma presents a different pathophysiological pattern than common tanning, ephelides and solar lentigines. A study on long wavelength UVA and visible light (violet and blue) identified a delayed pigmentation similar to melasma, and it was not identified among lighter phototypes [53–55]. The evolutive gain of melanogenesis promoted by non-ionizing radiation in darker skin is still not understood, but can support the relapsing of melasma despite adequate UVB and short wavelength UVA photoprotection [56].

There are histopathological similarities found in skin with melasma and dark skin. African skin has large non-aggregated melanosomes distributed through all layers of the epidermis with an increased number in the basal layer [57, 58]. The *stratum corneum* from black skin has less lipids and the dermis has the same thickness as Caucasian skin, however fibroblasts and macrophages are larger, more numerous, and hyperactive [57, 59, 60]. In an analogous way, the histopathological characteristics of highly pigmented skin, such as more mature melanosomes, greater dermic cellularity and reduced lipid layer are also present in patients with melasma. Moreover, after sun exposure, skin with melasma develops a more intense pigmentation than adjacent skin, as a localized darker phenotype [2, 61–64].

There was no information on melasma prevalence according to social strata or income levels. This is the first study to explore its prevalence across educational levels. Lower education is associated with lower socioeconomic status, higher overall mortality, less information regarding disease prevention, and less concern with personal health. Social and educational status can be considered as a proxy indicator for these factors that can be a confounding in the association with melasma and ancestry [65]. Patients with melasma and low schooling level could be less adept at sun protection, probably due to the lack of information on prevention and the cost of sunscreen products. They can be more exposed to the sun in the course of their daily life, as they work in jobs which require less instruction but more exposure to UVR.

Our study also showed that the number of facial topographies affected by the disease is lower in relation to education level. Also, schooling is associated with lower African and Amerindian ancestral components, in contrast with European ancestry. However, low educational level and African ancestry were independently associated with melasma. In addition to educational level, further studies need to evaluate the association between family income and melasma and its severity.

Observational studies are subject to memory and information bias, which in this case could have occurred in family history, use of medication and daily sun exposure. It is believed that there were similar imprecision levels between cases and controls which minimises their impact in final results.

Generalisation of this study is difficulted due to the fact that cases and controls were recruited from a public dermatologic service in the interior of Brazil, however, group homogeneity in relation to patient geographic and social origin, guarantees comparability between them. Similarly, the exclusion of Asian individuals and extreme phototypes (I & VI)-specifically associated with European and African ancestry, maximised the exploration of cases and controls with a higher genetic admixture, strengthening the internal validity of our results.

This investigation must be further pursued in patients with extrafacial melasma, men, and other populations with different genetic ancestral components, such as Middle Eastern, East Asian and populations from Oceania. Additionally an exploratory study on the mosaicism of the pigmentary system, especially in genes reported as related to ancestry, is warranted.

## Conclusions

Facial melasma was independently associated with African ancestry in a highly admixed population.

## Additional files

Additional file 1: Table S1. List of INDELs. (DOCX 22 kb)
Additional file 2: Table S2. Sensitivity analysis of data without imputation (*n* = 180). (DOCX 16 kb)
Additional file 3: Table S3. Correlation coefficient (Spearman’s rho) between ancestry component and skin phototype, education level or facial topographies affected (*n* = 119). (DOCX 14 kb)
Additional file 4: Table S4. Number of facial topographies affected by melasma according to schooling and skin phototypes (*n* = 119). (DOCX 14 kb)
