# Supplementary material for: African ancestry is associated with facial melasma in women: a cross-sectional study
Source: BMC Med Genet. 2017 Feb 17;18:17. doi: 10.1186/s12881-017-0378-7 (PMC5316149; doi:10.1186/s12881-017-0378-7)
Supplement: Additional file 1: Table S1. — List of INDELs. (DOCX 22 kb) [file 12881_2017_378_MOESM1_ESM.docx]

**Supplementary table 1.** List of INDELS.

| **Num. rs** | **Marker** | **Crom.** | **Position** | | | | **Alele1/Alele2** | | | **Amplicon** | | | **Primer** | | **DYE** |
| --- | --- | --- | --- | --- | --- | --- | --- | --- | --- | --- | --- | --- | --- | --- | --- |
| rs2307922 | MID1726 | 1 | | 38178337 | | [-/caagaactataat] | | | 243-256 | | | F- 5' GTCCAAATGCACCACAATCTT 3'  R- 5' TTT GTA CTA CGG CAC ATT AAG AGG 3' | | PET | |
| rs2308261 | MID2070 | 1 | | 59104261 | | [-/ttaca] | | | 305-312 | | | F- 5' TGT TGG GAT TTG AAC TCT TGATTA G 3'  R- 5'CAT ATC TCC ATT CTC CCA TAT CAT C 3' | | NED | |
| rs2307582 | MID1386 | 1 | | 221067572 | | [-/aaactattcatttttcaccct] | | | 327-348 | | | F- 5' ACA AAG AGA ACA GCC TCC AGA C 3'  R- 5' TAA TCA ATC CCC AGA CAA CTC C 3' | | VIC | |
| rs25574 | MID350 | 2 | | 205324392 | | [-/tta] | | | 102-105 | | | F- 5' CTT GGA GAT GTA CCA GGA AAA TG 3'  R- 5' AAG TGG GTG TTT CCA ATC ATT C 3' | | PET | |
| rs2308203 | MID2011 | 2 | | 103711267 | | [-/ctaga] | | | 192-197 | | | F- 5' TGAGTAGGCCAATAAGAACATCC 3'  R- 5' TTA CTT ACG CTT CGG GTT CAT T 3' | | PET | |
| rs16653 | MID216 | 2 | | 185153341 | | [-/tatt] | | | 226-231 | | | F- 5' CTG TAT TTG GTC ATT CAA GCT GTT 3'  R- 5' CTG TCC ACT TGG GTC TCA AAA T 3' | | NED | |
| rs2308205 | MID2013 | 2 | | 192427899 | | [-/ttgaagattagtttattcatcacaatg accttagactac] | | | 280-326 | | | F- 5'CAG CAG ATT CAA GAA TGT CAC C 3'  R- 5'ATT CCA TGC ATG TGT AAG CAG A 3' | | PET | |
| rs2307828 | MID1632 | 2 | | 74780497 | | [-/gtggtata] | | | 328-336 | | | F- 5'CCAAAGTGGAGAGAAACAGAGG3'  R- 5'GCAACCCTCACTCTAGATCACC3' | | 6-FAM | |
| rs2307981 | M1D1785 | 3 | | 12001549 | | [-/ttg] | | | 95-98 | | | F- 5' CGGGTGTGCATTCTACATTCT 3'  R- 5' GGA GGA TGA ATA TGG TGG TCT C 3' | | NED | |
| rs2307644 | MID1448 | 3 | | 189561118 | | [-/atac] | | | 162-165 | | | F- 5' GAT CTC CTC TGA TCC TGA TTT TG 3'  R- 5' CAT GGA TAA CTC TGT GGA TGG A 3' | | 6-FAM | |
| rs4183 | MID17 | 3 | | 3131718 | [-/taac] | | | 197-201 | | | F- 5'ACT GCA ACC CTC CAA GTA ATG T 3'  R- 5' GAT CCC AGA CAC TGA AGA TGA A 3' | | | NED | |
| rs1611095 | MID1013 | 5 | | 122973840 | [-/ccag] | | | 99-103 | | | F- 5'CCTAATGCCTTTTTCTGTTCCA3'  R- 5'TGTTTAGCTTCCTGGACTGGTT3' | | | PET | |
| rs1611070 | MID988 | 5 | | 109678924 | [-/cttt] | | | 149-153 | | | F- 5' CAC AAT AGC CAT GTT TGC ATC T 3'  R- 5' CCT TTC TCC TGA TTC ACC AAA T 3' | | | PET | |
| rs1610875 | MID789 | 5 | | 9286377 | [-/agag] | | | 161-165 | | | F- 5' CAGAGTAGAAATTGGCTTCCTCA 3'  R- 5' GAT GCT ATC AGT CTT GCT AAT GGA 3' | | | NED | |
| rs2307799 | MID1603 | 5 | | 66724153 | [-/ttgt] | | | 234-238 | | | F- 5' AAA AGA CTG GAG GTT CCT TTT GA 3'  R- 5' GTG CAA ATA AAA CGC AAC AGA A 3' | | | VIC | |
| rs2067128 | MID1039 | 5 | | 33862409 | [-/atggttcattgtgctgtgcctg tgacccttgcaaggtggt] | | | 211-251 | | | F- 5' GCG TTT CAT CTC TTT GGG TTA G 3'  R- 5' CTG TTC TTA GCA GTG GGC TTT C 3' | | | VIC | |

**Continue.**

| **Num. rs** | **Marker** | **Crom.** | **Position** | **Alele1/Alele2** | | **Amplicon** | | **Primer** | **DYE** |
| --- | --- | --- | --- | --- | --- | --- | --- | --- | --- |
| rs1610941 | MID856 | 5 | 62283289 | [-/gaatcttctaatcacaacgaagaaga] | 256-282 | | F- 5' TTA ATT CTT GAG GGC AGA GGA G 3'  R- 5' TAT TGT GCT CAT TTT CTG GGT C 3' | | 6-FAM |
| rs140762 | MID473 | 6 | 11581161 | [-/aaatgtaa] | 83-91 | | F-5' GCA ACA ACC AAG ACA AGG ATT 3'  R-5' AAA ATG TTA AGC CTC CCC TGT 3' | | 6-FAM |
| rs1160850 | MID591 | 6 | 22854507 | [-/ttaa] | 114-118 | | F- 5'ATT GCC ATC ACT GTA ATC TTG GT 3'  R- 5'AAT TAG AGT AGA AAA GCA GCT TCC A 3’ | | 6-FAM |
| rs140847 | MID558 | 9 | 12549927 | [-cgtt] | 311-315 | | F- 5'TAAGTGAGATAATAAATGCGCTTGG3'  R- 5'GTGGATACCTGTGGAGAGAAGAAA3' | | 6-FAM |
| rs2067186 | MID1098 | 10 | 72466651 | [-/aatcaatatt] | 340-349 | | F- 5' TCC TTT CAG GTA TCA GTC CCC 3'  R- 5' TGG AGA GAA GTG GGT AGA TTT GA 3' | | NED |
| rs2307976 | MID1780 | 11 | 35294648 | [-/gaa] | 104-107 | | F- 5' AGG AAA CGG CAC TTA TGA CTT C 3'  R- 5' CGC AGA TAT TAG CCT GAC CTT C 3' | | NED |
| rs2307666 | MID1470 | 11 | 62055890 | [-/gttac] | 133-139 | | F- 5' AGT CTG ACC CTT CAT AAG CAA GA 3'  R- 5' ATC CCA AAC AAC CAC ATA GGA G 3' | | NED |
| rs2308144 | MID1952 | 15 | 40049495 | [-/tcca] | 96-100 | | F- 5' TTT GCT CCC ACA GCA TTA GTC 3'  R- 5' AGT AAA CAA ACG GCT GCA AGA 3' | | 6-FAM |
| rs2067259 | MID1172 | 16 | 2410877 | [-/ggcct] | 113-118 | | F- 5' CTC CCA AAG TGC TGG GAT TAC 3'  R- 5' CAGCTTGTCTTGGTCAACTGC 3' | | VIC |
| rs1610864 | MID778 | 16 | 68144755 | [-/aat] | 197-200 | | F- 5' GTG ACA GGC TTT ATA GGC CAA C 3'  R- 5' TTT ACC TGC TGT GGA CTT GAT G 3' | | VIC |
| rs2067270 | MID1183 | 16 | 43065720 | [-/tcag] | 164-168 | | F- 5'AATGGACTGTGATTTTGGATTTG3'  R- 5'ATGAAGTTGACATGTGGGAATG3' | | PET |
| rs16710 | MID273 | 17 | 5404941 | [-/caac] | 137-141 | | F- 5'GGTGTGATCAATTCCAACTGC 3'  R- 5'AAA GGA AAT CAC GTC TAA GTG ACC 3' | | 6-FAM |
| rs25549 | MID325 | 17 | 54492124 | [-/aatg] | 209-211 | | F- 5'TTAGGTATACGGCAGAGGATGG 3'  R- 5'CATGCACCTCCTTGGTTATGT 3' | | PET |
| rs2307587 | MID1391 | 17 | 17176824 | [-/tacatatatcaatctataac] | 218-238 | | F- 5'AATTCCCAGGGATAACCACTCT3'  R- 5'CCTGGCTCATAATTGGGTTTT3' | | NED |
| rs16383 | MID93 | 22 | 25854689 | [-/aga] | 146-149 | | F- 5' TGA CCC CAG TTA CTA ATG AAA ACC 3'  R- 5' CAC AGC TTT TTC TCC AGT GTT G 3' | | VIC |

**Continue.**

| **Num. rs** | **Marker** | **Crom.** | **Position** | **Alele1/Alele2** | **Amplicon** | **Primer** | **DYE** |
| --- | --- | --- | --- | --- | --- | --- | --- |
| rs140864 | MID575 | 1 | 34666090 | [-/ttc] | 175-179 | F- 5' AAC TGG TCA AAA TCT GCT CCA T 3'  R- 5' TAC GTG GAC CTA GCA TTC CTC T 3' | NED |
| rs2307659 | MID1463 | 1 | 77934148 | [-/ttgt] | 293-297 | F- 5' CAG AAG TAG GCT GAC TGG TGT TT 3'  R- 5' CAG AGT ATC TGG GGC TTT TCA C 3' | 6FAM |
| rs1160876 | MID625 | 2 | 31342236 | [-/cat] | 115-118 | F- 5' TAC GGC TAG GCT ACA AGA CCT C 3'  R- 5' CACAAGGAGACAGAACTTCATCA 3' | PET |
| rs140770 | MID481 | 2 | 63724924 | [-/caggtac] | 283-290 | F- 5' AGA TTT GAT AGA AAT GCA GGA ACC 3’  R- 5' GTA GTT GGG GAA GAG TGC AGA C 3’ | NED |
| rs16343 | MID51 | 4 | 18100554 | [-/tttat] | 273-278 | F- 5' TTG ATT GGG TGA GCA TTA TTT 3'  R- 5' TTT GCA TCT GTA GAC TGG TTG G 3' | VIC |
| rs2067353 | MID1271 | 5 | 105719772 | [-/atttt] | 144-150 | F- 5' GTA TTA AGG TTT CCG GCC TCA C 3'  R- 5' AAGATGAGAAAGCAGGTTTGGA 3' | NED |
| rs2067263 | MID1176 | 5 | 52479514 | [-/aat] | 149-152 | F- 5' GCT TAC CTA GCA GGG TTG TTG T 3'  R- 5' AAACTCCCAGTCCAAGGTGATA 3' | VIC |
| rs2067141 | MID1052 | 5 | 88239324 | [-/acaa] | 212-216 | F- 5'GGCGTAAAATTAAATACACAGGACA3'  R- 5'GGCTGCATCTATAAGACACTTCATT3' | NED |
| rs1611106 | MID1025 | 5 | 18803448 | [-/gttaa] | 260-265 | F- 5'CACCGCTTCTTTGTATGGATAGTT3'  R- 5'ATGCTTATTGAGGAAGCAGTCATAG3' | PET |
| rs16635 | M1D196 | 6 | 100433948 | [-cat] | 112-115 | F- 5' AAC CAA GTT CTA GCC ATA TGG AAC3’  R- 5' AAA GCT TCA GTG AAT TCC AAG G 3' | VIC |
| rs2308115 | MID1923 | 6 | 13185905 | [-/tga] | 134-138 | F- 5' CAC TAC TGA GAT GCT TTC ACC AA 3'  R- 5’ CCT TTC CTG TAC GTG CTT CTT T 3’ | 6FAM |
| rs2307912 | MID1716 | 6 | 84275361 | [-/aga] | 182-186 | F- 5' TTT GTG TGT GTG TGT GTG TGT G 3'  R- 5' ACCTCAGCATCACCAGAAGAGT 3' | VIC |
| rs140783 | MID494 | 6 | 8388809 | [-/cgatgcatct] | 227-236 | F- 5' AGACACATGGAGGAAAGACCAT 3'  R- 5' TGT TAC GGA AAT CAC AAG AAC G 3' | NED |
| rs1610996 | MID913 | 6 | 39865711 | [-/aaagtgaaattca] | 331-344 | F- 5' CTA GAA TGT CAA GAC CTG GGA TG 3'  R- 5' TGG CCT TTG ATT GGA TAT GAG 3' | NED |

**Continue.**

| **Num. rs** | **Marker** | **Crom.** | **Position** | **Alele1/Alele2** | **Amplicon** | **Primer** | **DYE** |
| --- | --- | --- | --- | --- | --- | --- | --- |
| rs16654 | MID217 | 7 | 140766058 | [-/ata] | 171-174 | F- 5' AAA GGC AGA TTT AGG GAC CAC 3'  R- 5' GGA TGA AGG GGA AAA AGT ATG A 3' | PET |
| rs1160871 | MID619 | 7 | 28158757 | [-/tctt] | 198-201 | F- 5' GAC TCT TTT CCT TGA CCC TTC A 3'  R- 5'GAG AGG TAG AGG GAG TTT GGG 3' | 6FAM |
| rs140765 | MID476 | 9 | 19639579 | [-/aga] | 167-170 | F- 5' CAT TGA TGT CTA TTT GCC TTT GAG 3'  R- 5' TTC CTA GGG CTG GAG TTA CTG A 3' | 6FAM |
| rs1160910 | MID682 | 9 | 67940083 | [-/aaag] | 173-176 | F- 5' CTC CAT TCT TTC AGC CAA CTC T 3'  R- 5' CAA AGC ACT TCA TGG AAT TAA GG 3' | VIC |
| rs1160894 | MID660 | 10 | 17295939 | [-/cat] | 109-112 | F- 5' AAA GGC CAT GCC AAT AAT TTT C 3'  R- 5' ATG GCA AGTCAT TCA ATC CTT C 3' | NED |
| rs140857 | MID568 | 13 | 74794171 | [-/aca] | 201-204 | F- 5' TTT AGT GTG CCT CCT TCA AAC A 3'  R- 5' GGG TGA GTT GTT GAG TGT GAG T 3' | PET |
| rs2307553 | MID1357 | 14 | 60159869 | [-/tgac] | 95-99 | F- 5' TTA TAC CTG CAA AGT GGG CAT T 3'  R- 5'GTTCCAGCAAACAGAACACAAG 3' | 6FAM |
| rs2307880 | MID1684 | 14 | 18692312 | [-/aggtgg] | 157-163 | F- 5'GTGATGTTTTCACAAGCAGGAA 3'  R- 5'TCA TCC ATG TCT ATG TCT TCA GC 3' | 6FAM |
| rs16416 | MID132 | 15 | 3527138 | [-/acag] | 190-194 | F- 5' AAT GAG ATT GCC AGC TTT CCT A 3'  R- 5' CCA CCC ACA AAA TTG GAC ATA 3' | NED |
| rs1610902 | MID818 | 16 | 2415015 | [-/gtctggggagctgttctctacccc] | 222-245 | F- 5' AGA GCC AGT TAG AGG GAG GAT G 3'  R- 5' GAA CGA GAG TGC GAA CCA AAT 3' | 6FAM |
| rs2067271 | MID1184 | 16 | 68111678 | [-/tgtgcagatg] | 243-253 | F- 5' CGG TTC AGT CTT GAT TGT GCT A 3'  R- 5' TCC AAT TCT CTC CTG CAA AAC T 3' | PET |
| rs25546 | MID322 | 17 | 12704466 | [-/aaat] | 117-121 | F- 5'AAA ACC TTG TGG TCT GTA ATG AGG 3'  R- 5'CAG CCC TGG GTT ATT ATT CTT ATT T 3’ | NED |
| rs16712 | MID275 | 17 | 65454902 | [-/acact] | 210-215 | F- 5' TCT TTC TGC CAA TGA CTT CTA GC 3'  R- 5' ACA AAC TGA CTG ACT TCC ACG A 3' | 6FAM |
| rs2307554 | MID1358 | 5 | 35502077 | [-/agaatgacttcattctg] | 235-252 | F- 5' AAA AAC ACC CTT TGC TGA AGT C 3'  R- 5' ACA GAC GCC AGG AAT TTT CTA T 3' | VIC |
| rs16432 | MID152 | 20 | 2192651 | [-/cggcagtgg] | 255-263 | F- 5' CTC CCT GCT GAT ACC GTC TAT AA 3'  R- 5' GGA GGC AGA AGT CCA GAG ATA C 3' | 6FAM |
