# Supplementary material for: African ancestry is associated with facial melasma in women: a cross-sectional study
Source: BMC Med Genet. 2017 Feb 17;18:17. doi: 10.1186/s12881-017-0378-7 (PMC5316149; doi:10.1186/s12881-017-0378-7)
Supplement: Additional file 2: Table S2. — Sensitivity analysis of data without imputation (n = 180). (DOCX 16 kb) [file 12881_2017_378_MOESM2_ESM.docx]

**Supplementary table 2.** Sensitivity analysis of data without imputation (n=180).

| **Variables** | ***Multivariate analysis*** | |
| --- | --- | --- |
|  | **Odds Ratio (CI 95%)** | **p** |
| Age (years)* | 0.96 (0.91-1.01) | 0.10 |
| Skin phototype – N (%) |  | 0.09 |
| II | 1.00 (-) |  |
| III | 1.88 (0.71-4.95) |  |
| IV | 2.61 (0.92-7.36) |  |
| V | 0.60 (0.13-2.70) |  |
| Education level – N (%) |  | **0.03** |
| Elementary-Middle school | 4.96 (1.49-16.55) |  |
| High school | 1.24 (0.55-2.82) |  |
| College | 1.00 (-) |  |
| Family with melasma (first degree) – N (%) | 2.80 (1.28-6.15) | **<0.01** |
| Age of menarche (years)* | - | - |
| Time using hormonal contraception (years)^#^ | 1.03 (0.98-1.08) | 0.26 |
| Daily regular sun exposition – N (%) | 1.19 (0.51-2.80) | 0.70 |
| Pregnancy history – N (%) | 2.17 (0.85-5.60) | 0.11 |
| Psycotropic drugs (regular use) – N (%) |  |  |
| Antidepressant | 2.22 (0.13-36.67) | 0.58 |
| Anxiolytic | - | - |
| Genetic ancestry (%)^#^ |  |  |
| European component | - | **-** |
| Amerindian component | - | - |
| African component | 1.05 (1.01-1.09) | **<0.01** |
| Interactions |  |  |
| Antidepressant * Family with melasma | 6.15 (1.13-33.37) | **<0.01** |
| Antidepressant*Pregnancy history | 0.14 (0.01-1.35) | 0.09 |

p (overall model)<0.01; p (constant)<0.05; Hosmer-Lemeshow test: p=0.77; Correct classification: 73%; R^2^ (Nagelkerke): 0.38.

* mean (st deviation); ^#^ median (p25-p75).
