# Supplementary material for: African ancestry is associated with facial melasma in women: a cross-sectional study
Source: BMC Med Genet. 2017 Feb 17;18:17. doi: 10.1186/s12881-017-0378-7 (PMC5316149; doi:10.1186/s12881-017-0378-7)
Supplement: Additional file 3: Table S3. — Correlation coefficient (Spearman’s rho) between ancestry component and skin phototype, education level or facial topographies affected (n = 119). (DOCX 14 kb) [file 12881_2017_378_MOESM3_ESM.docx]

**Supplementary table 3.** Correlation coefficient (Spearman´s rho) between ancestry component and skin phototype, education level or facial topographies affected (n=119).

|  | European | African | Amerindian |
| --- | --- | --- | --- |
| Education (n=238) | 0.236* | -0.241* | -0.160* |
| Skin phototype (n=238) | -0.426* | 0.407* | 0.177* |
| Topographies affected (n=119) | -0.230* | 0,178** | 0.146 |

* p<0.01; ** p=0.05
