# Supplementary material for: African ancestry is associated with facial melasma in women: a cross-sectional study
Source: BMC Med Genet. 2017 Feb 17;18:17. doi: 10.1186/s12881-017-0378-7 (PMC5316149; doi:10.1186/s12881-017-0378-7)
Supplement: Additional file 4: Table S4. — Number of facial topographies affected by melasma according to schooling and skin phototypes (n = 119). (DOCX 14 kb) [file 12881_2017_378_MOESM4_ESM.docx]

**Supplementary table 4.** Number of facial topographies affected by melasma according to schooling and skin phototypes (n=119).

| **Variables** | **Affected topographies*** | **p-value** |
| --- | --- | --- |
| Education level |  | <0.01 |
| Elementary | 4 (2.3) |  |
| High school | 3.4 (1.7) |  |
| College | 2.4 (1.1) |  |
| Phototype |  | 0.11 |
| II | 2.6 (1.3) |  |
| III | 3.2 (2.0) |  |
| IV | 3.8 (2.1) |  |
| V | 2.8 (1.1) |  |

* mean (standard deviation)
